# *Melanaphis* *sorghi* (Hemiptera: Aphididae) Clonal Diversity in the United States and Brazil

**DOI:** 10.3390/insects13050416

**Published:** 2022-04-28

**Authors:** Karen Harris-Shultz, John Scott Armstrong, Geraldo Carvalho, Jurandir Pereira Segundo, Xinzhi Ni

**Affiliations:** 1 Crop Genetics and Breeding Research Unit, USDA-ARS, 115 Coastal Way, Tifton, GA 31793, USA; xinzhi.ni@usda.gov; 2 Wheat, Peanut and Other Field Crops Research Unit, USDA-ARS, 1301 N. Western Rd, Stillwater, OK 74075, USA; scott.armstrong@usda.gov; 3 Helix Sementes e Mudas LTDA, Rodovia MT-242, KM 19, Zona Rural, Sorriso 78890-000, Brazil; geraldo.carvalho@agroceres.com or; 4 Helix Sementes e Mudas LTDA, Rua Arnaldo Luiz de Oliveira, 75 Bela Vista, Patos de Minas 38703-240, Brazil; jurandir.segundo@agroceres.com

**Keywords:** super-clone, *Melanaphis sacchari*, microsatellite, simple sequence repeat, sorghum aphid, multilocus lineage, sugarcane aphid

## Abstract

**Simple Summary:**

*Melanaphis sorghi* has been a perennial economically important pest to U.S. sorghum since 2013. Previous research has shown its recent infestation on sorghum has been spreading as a super-clone, a highly abundant clone that is distributed over a large geographic area and persists over time, in the U.S. To continuously monitor the genotypes present in the U.S. and to determine the genotype present in Brazil on sorghum, *Melanaphis* spp. were collected in 2019 and 2020. Genotyping of aphid samples with microsatellite markers revealed that the super-clone predominated in the U.S. in 2019 and 2020 and Brazil in 2020. Thus, the *M*. *sorghi* super-clone remains in the U.S. on sorghum, Johnsongrass, and giant miscanthus and is present in Brazil on sorghum.

**Abstract:**

*Melanaphis sorghi* (Hemiptera: Aphididae), are an economically important pest to sorghum in the Americas. Previous studies have found that a super-clone that belongs to multilocus lineage (MLL)-F predominated in the U.S. from 2013 to 2018 and uses multiple hosts besides sorghum. In contrast, previous studies found that aphids in South America belong to MLL-C, but these studies only examined aphids collected from sugarcane. In this study we sought to determine if the superclone persisted in the U.S. in 2019–2020 and to determine the MLL of aphids found on sorghum in the largest country in South America, Brazil. *Melanaphis* spp. samples (121) were collected from the U.S. in 2019–2020 and Brazil in 2020 and were genotyped with 8–9 *Melanaphis* spp. microsatellite markers. Genotyping results showed that all samples from the U.S. in 2019 and Brazil in 2020 had alleles identical to the predominant superclone. Of the 52 samples collected in the U.S. in 2020, 50 samples were identical to the predominant super-clone (multilocus lineage-F; *M. sorghi*), while two samples from Texas differed from the super-clone by a single allele. The results demonstrated that the super-clone remains in the U.S. on sorghum, Johnsongrass, and giant miscanthus and is also present on sorghum within Brazil.

## 1. Introduction

*Melanaphis sorghi* have been an economically important pest to U.S. sorghum (*Sorghum bicolor*) since their discovery on grain sorghum near Beaumont, TX in the late summer of 2013 [1]. This invasive parthenogenic aphid pest has spread rapidly, and within two years, it has infested sorghum crops in 17 U.S. states, which accounted for 98% of the total sorghum production in the U.S. [2]. This recent invasive aphid species has been found to feed on all types of sorghum, Johnsongrass (*Sorghum halepense*), sudangrass (*Sorghum verticilliflorum*), energycane (*Saccharum* spp.), Columbus grass (*Sorghum almum*; a hybrid between sorghum and Johnsongrass), and giant miscanthus (*Miscanthus* x *giganteus*) [1,3,4,5]. Interestingly, this new *M*. *sorghi* invader reproduces poorly on sugarcane [5]. 

*M*. *sorghi* damages sorghum by feeding on the sap from phloem tissue of the leaves, stems, and panicles causing a loss of plant nutrients and sugars [6,7]. Aphid populations can reach tremendous numbers (exceeding 10,000 aphids per plant) on sorghum and aphid feeding can result in leaf chlorosis, leaf necrosis, stunted growth, delayed or prevention of panicle emergence, and plant death [2,7,8,9,10]. Additionally, *M*. *sorghi* also causes a reduction in photosynthetic efficiency due to its secretion of honeydew that causes a sooty mold to buildup on the sorghum leaves [7]. This sticky honeydew also causes problems at harvest as combines become clogged and grain may be expelled from the combine discharge [1]. Yield decline on susceptible grain sorghum hybrids ranged from 50–100% in infested fields [6,10].

Prior to the U.S. invasion in 2013, *Melanaphis* spp. diversity was examined worldwide, and aphids were collected on sugarcane and Johnsongrass in 2007 from Louisiana and Hawaii [11]. These aphids from the U.S. were classified as multilocus lineage (MLL)—D. *Melanaphis* spp. diversity in the U.S. has been examined from 2013–2018. Harris-Shultz et al. [12] found that *M*. *sorghi* collected on sorghum in 2015 from 17 locations across seven states and one U.S. territory were predominantly one “superclone”. Nibouche et al. [13] collected *Melanaphis* spp. samples from 2013–2017 from primarily North America and the Caribbean and found that this new invasive aphid pest on sorghum had not been identified previously in their worldwide study and classified this “superclone” as belonging to MLL-F. In the U.S. since 2013, the MLL-F “superclone” (*M. sorghi*) has been found feeding on sorghum, sugarcane, Johnsongrass, giant miscanthus, and Columbus grass whereas aphids classified as MLL-D have been found feeding only on sugarcane [13,14]. Recently, MLL-C, and D aphids have been classified as *M. sacchari*, MLL-A and F aphids have been reclassified as *M. sorghi,* and MLL B and E aphids have not been assigned a species designation although SSR and EF1-α sequence data suggest both belongs to *M. sorghi* [15]. Although the Entomological Society of America has not adopted this change, the common names have also been altered and aphids that are classified as *M. sacchari* have a common name as sugarcane aphids and aphids classified as *M. sorghi* are now called sorghum aphids [15].

Diversity of *Melanaphis* spp. in South America has been previously examined. Samples (131) from Brazil, Ecuador, and Columbia collected from 2008–2009 on sugarcane all belonged to MLL-C [11]. In 2016, four *Melanaphis* spp. samples were collected from Peru on sugarcane and were also found to be MLL-C [13]. In a personal communication, three samples collected from Brazil in 2020 were reported to be *M. sorghi* but the host plant (sorghum or sugarcane) from which it was collected or the MLL it was assigned was not reported [15]. Diversity of *Melanaphis* spp. on sorghum in South America warrants further investigation.

Seed companies have responded to the *M. sorghi* outbreak by providing sorghum producers with hybrids that express antibiosis or tolerance (and in some cases both) [16] to the MLL-F “superclone” [17]. To date, *Melanaphis* spp. worldwide have been classified into six MLL [13] and with global trade and prevailing jet streams it is possible to have a new MLL enter the U.S. Changes in the predominant clonal genotype in the U.S. is also possible especially with the use of insecticides listed within the same Insecticide Resistance Action Committee (IRAC) group to aide in the selection of resistant genotypes [18]. The sorghum industry could be severely impacted if a new *Melanaphis* spp. biotype or pesticide resistant genotype spreads throughout the sorghum growing region. The sorghum industry suffered when new economically important greenbug biotypes overcame host resistance from the 1970s–1990s [as reviewed in 18]. In this study we examined *Melanaphis* spp. diversity in the U.S. from 2019 to 2020 to confirm if the “superclone” was still present as the dominant genotype. We also examined *Melanaphis* spp. diversity in Brazil on sorghum to determine the MLL that was present on sorghum in 2020.

## 2. Materials and Methods

Aphid samples were collected from sorghum, Johnsongrass, and giant miscanthus in 2019 and 2020 (Supplemental Table S1). For the samples from the U.S., for large fields, 2–3 samples were collected per field where a sample consists of 4–5 infested leaves. Samples were mailed overnight to Tifton and a pooled sample and a clonal sample was created from each mailed sample. The pooled sample consisted of aphids filled to the 0.5 mL mark in a 2 mL tube. For the clonal sample, a single aphid was moved to a benzimidazole agar plate [19,20] containing either cut sorghum or Johnsongrass leaves as the food source. Aphids were reared on the agar plates until approximately 0.1 mL of aphids were obtained. The aphids were then moved into a 2 mL tube and were immediately frozen in a −80 °C freezer. Additionally, some of the *Melanaphis* spp. samples were shipped frozen on dry ice. For these samples, the microcentrifuge tubes were placed in the −80 °C freezer until DNA extraction.

For the samples from Brazil, aphids were shipped in 90% alcohol. The day prior to DNA extraction, the alcohol was removed and 350 µL of Lysis buffer A (Thermo Fisher Scientific, Waltham, MA, USA) was added to each tube. Samples were placed overnight at 4 °C and the following morning a DNA extraction was performed. 

For each sample, four Zn-plated BBs (Daisy Outdoor Products, Rogers, AR, USA) was added into each 2 mL tube and the tubes were placed in liquid nitrogen. Samples were ground using a vortexer by repeatedly taking the samples out of the liquid nitrogen, grinding for less than 10 s, and then placing the tube back into the liquid nitrogen. Samples were ground into a fine powder. DNA was extracted using a GeneJET Plant Genomic DNA Purification kit (Thermo Fisher Scientific) following the manufacturer’s recommendations, except aphids were used instead of plant tissue and one elution was performed. The purified DNA was quantified using a NanoDrop 2000c (Thermo Fisher Scientific), and DNA quality was determined by visualization of the DNA on a 1% agarose gel.

DNA fragments containing Simple Sequence Repeats (SSR) were amplified from each *Melanaphis* spp. sample by performing 10 µL PCR reactions. The *Melanaphis* spp. SSR markers used have been previously published [21]. For the 2019 and 2020 U.S. aphid samples, the SSR markers CIR-Ms-B09, CIR-Ms-D02, CIR-Ms-E01, CIR-Ms-G08, CIR-Ms-G403, CIR-Ms-C08, CIR-Ms-G01, CIR-Ms-E03, and CIR-Ms-G02 were amplified. The 2020 aphid samples from Brazil were amplified with the same SSR markers except CIR-Ms-B09. Each reaction contained 2 µL of 5× Colorless GoTaq Flexi buffer (Promega, Madison, WI, USA), 1 µL of 25 mM MgCl_2_, 0.8 µL of 2.5 mM dNTP mix, 1.8 µL of 1 μM M13 primer (M13-TGTAAAACGACGGCCAGT) 5′ labeled with FAM or HEX, 0.5 µL of 1 μM forward primer with a 5′ M13 tag, 2 µL of 1 μM reverse primer, 0.04 μL of GoTaq Flexi DNA polymerase (Promega), 0.86 µL of water, and 1 μL of sample DNA diluted to 2.5 ng^-µL^ The template arrays (three in total: 2019 USA, 2020 USA, 2020 Brazil) contained all aphid samples as well as the controls Armstrong 1, Armstrong 48, Bellflower1, Brewer4, and at least two no template controls (water only). The thermocycler conditions were an initial denaturation at 94 °C for 3 min, 39 cycles of 94 °C for 30 s, 50 °C for 1 min, 72 °C for 1 min and 10 s, and a final elongation step at 72 °C for 10 min. PCR amplicons were then diluted with 20 µL of molecular biology grade water. To load the PCR fragments onto the SeqStudio Genetic Analyzer (Thermo Fisher Scientific, Waltham, MA, USA), each sample contained 8.5 µL of Hi-Di formamide (Thermo Fisher) and 0.5 µL of GeneScan 500 ROX dye size standard, and 1 µL of diluted PCR sample. Samples were denatured on a thermocycler using 94 °C for 5 min, then loaded onto the SeqStudio, and run using the default program ”fragment analysis”. The fragment data were analyzed using GeneMapper software version 6 (Thermo Fisher Scientific).

Fragments were coded for the program NTSYSpc [22] where a ”0” is the absence of a band, ”1” is the presence of a band, and “9” is a missing fragment due to a failed reaction. For each sized fragment, this coding was used for each sample. Genetic similarity between each pair of samples was calculated using the SIMQUAL module using the DICE coefficient of similarity [23]. An unweighted pair-group method using arithmetic averages (UPGMA) dendrogram was created from the similarity matrix by using the SAHN module in NTSYSpc.

## 3. Results

**Figure 1:**
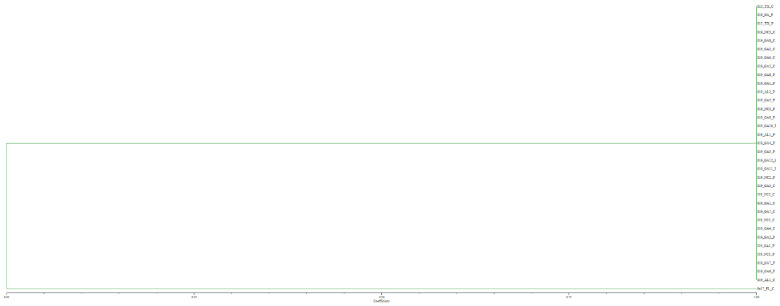
Unweighted pair group method with arithmetic mean dendrogram of 2019 *Melanaphis* spp. samples collected on sorghum and Johnsongrass from seven United States cities and genotyped using nine simple sequence repeat markers. All the *Melanaphis* samples collected in the United States in 2019 were found to be MLL-F (*Melanaphis sorghi*).

Genotyping of the 2019 U.S. *Melanaphis* spp. samples collected on sorghum and Johnsongrass from seven cities encompassing three states with nine SSR markers, generated 26 alleles (Supplemental Table S2). This analysis revealed that all 2019 samples (N = 30) had alleles that were identical to S13_TX_C, the sample used to represent the predominant genotype of MLL-F (Figure 1). The sample Su17_FL_C, the sample used to represent the MLL-D lineage grouped by itself.

**Figure 2:**
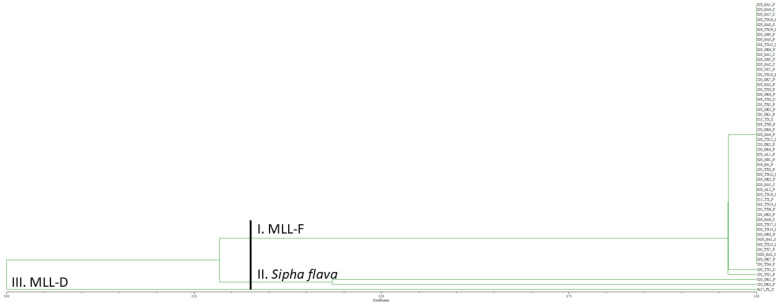
Unweighted pair group method with arithmetic mean dendrogram of 2020 *Melanaphis* spp. samples collected on giant miscanthus, sorghum, and Johnsongrass from 13 United States cities and genotyped using nine simple sequence repeat markers. *Sipha flava* was used as an outgroup. All the *Melanaphis* samples collected in the United States in 2020 were found to be MLL-F (*Melanaphis sorghi*). MLL, multilocus lineage.

Genotyping of the 2020 U.S. *Melanaphis* spp. samples collected on giant miscanthus, Johnsongrass, and sorghum from 13 cities encompassing six states with nine SSR markers generated 41 alleles (Supplemental Table S2). Of the 54 U.S. aphid samples collected in 2020, 52 were *Melanaphis* spp. samples. All 52 of the *Melanaphis* samples grouped with S13_TX_C, the MLL-F control (Figure 2). Within this group, 50 of the 2020 U.S. samples had identical alleles to S13_TX_C and two samples from Texas differed from S13_TX_C by one allele. The sample representing MLL-D, Su17_FL_C, formed a group by itself. Two samples from Oklahoma, S20_OK1_P and J20_OK2_P grouped together, had markedly different allele sizes than the other samples, and are yellow sugarcane aphid, *Sipha flava*, samples (our outgroup). Furthermore, an error was observed for samples S20_OK1_P and J20_OK1_P as samples appeared to have been switched as S20_OK1_P groups with the yellow sugarcane aphid sample and J20_OK1_P groups with the *M. sorghi* samples.

**Figure 3:**
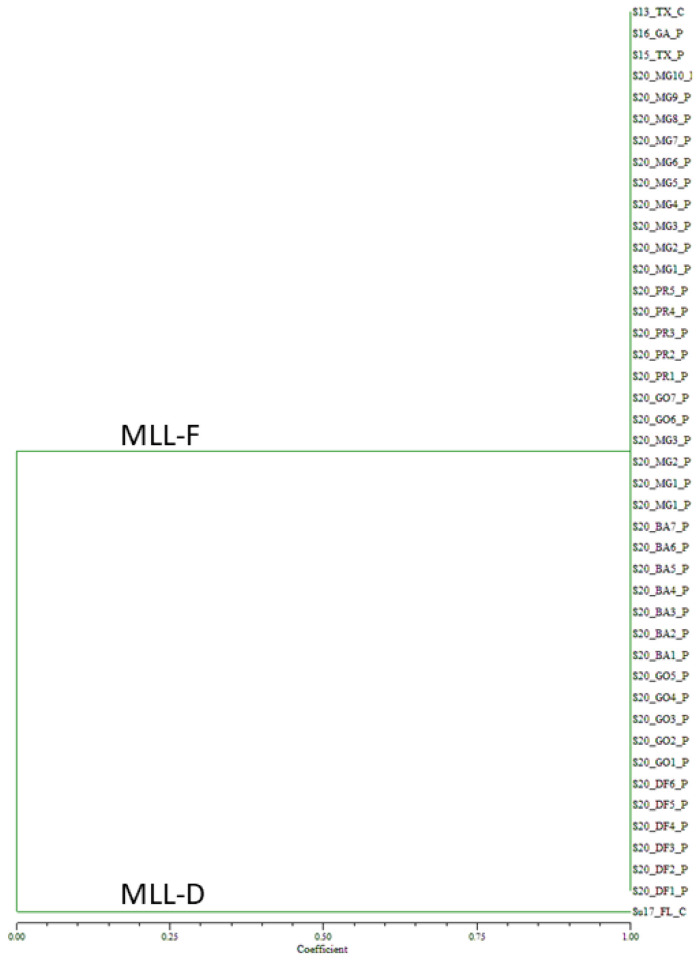
Unweighted pair group method with arithmetic mean dendrogram of 2020 *Melanaphis* spp. Samples collected on sorghum from ten Brazilian cities. Samples were genotyped using eight simple sequence repeat markers. All the samples collected in Brazil in 2020 were found to be MLL-F (*Melanaphis sorghi*). MLL, multilocus lineage.

Genotyping of the 2020 Brazilian samples collected on sorghum from ten cities encompassing four states and one federal district, and the controls with eight SSR markers generated 23 alleles (Supplemental Table S2). For the 39 Brazilian samples collected from 2020, all the samples showed identical alleles to the MLL-F control, S13_TX_C (Figure 3). In contrast, the MLL-D control sample, Su17_FL_C formed its own group.

## 4. Discussion

Genotyping of the 2019 and 2020 U.S. aphid samples on sorghum, Johnsongrass, and giant miscanthus and the 2020 Brazilian aphid samples on sorghum revealed that the MLL-F superclone is still predominant in the U.S. and is present in all 10 cities of Brazil examined (five states). MLL-F aphids have spread to all sorghum growing areas and persisted in the U.S. on sorghum and other hosts since 2013. This is the first identification of the MLL-F superclone in Brazil. An outbreak started in the state of Goias in 2020 and heavy aphid infestations were observed on sorghum (personal communication, Geraldo Afonso Carvalho). Besides the continental U.S. and Brazil, MLL-F aphids have also been detected in Mexico, Haiti, and Puerto Rico [11].

For the 2020 U.S. *Melanaphis* samples, two of the 52 samples (S20_TX1_C and J20_TX1_P) differed by one allele from the predominant genotype. This may be due to mutation or genotyping error. Both samples were from Texas, one collected from Johnsongrass next to a plot of sorghum and the other was collected from sorghum. Along the Mexico/US border, two crops of grain sorghum can be grown in the same year and Johnsongrass, a perennial grass species that is a pervasive weed in the U.S. that is also a very good host to *M. sorghi*, is present year-round. Furthermore, in Texas and Louisiana, MLL-D and MLL-F aphids are present as sugarcane and sorghum are grown in these areas but only asexual reproduction has been reported in the U.S. [2,24,25]. Southern regions (latitude ≤ 31° N) provide environments where *M. sorghi* can survive year-round. Many more generations of *M. sorghi* can be produced in southern environments than in more northern regions that experience harsher winters that kill the aphids. These southern locations may provide an ideal environment for the accumulation of mutations in *M. sorghi*. For example, Harrington [26] stated, in theory, a single asexual female aphid in just one growing season can produce 7.6 × 10^28^ offspring and aphid asexual lineages have been found to be mutating, even within a few generations [27,28,29]. Most of these mutations would not cause a change in phenotype but with time (more generations produced) and selection pressure, some would impact phenotype [29].

In agroecosystems, there is a trend for invasive asexual aphid lineages to have a few genotypes or super-clones as compared with species with cyclical parthenogenesis which have many unique genotypes [30]. The main variables allowing these aphids to thrive in these areas are obligate parthenogenesis, host availability, and phenotypic plasticity. The MLL-F *M. sorghi* super-clone in the U.S. and Brazil follows this trend as it does reproduce by obligate parthenogenesis, feeds on multiple species of which one, Johnsongrass, is a perennial invasive weed found from 55° N to 45° S in latitude and thrives in a wide range of environments. 

*Melanaphis sorghi* remains a critically important pest for all types (i.e., grain, forage, biomass, and sweet) of sorghum production. Despite natural enemies including Coccinellidae, Syrphidae, Chrysopidae, Hemerobiidae, Anthocoridae, and Hymenopterans being recruited to the aphid-infested sorghum, they cannot decrease aphid populations below economic threshold [2,31,32]. Genotyping of *Melanaphis* spp. from the U.S. in 2019 and 2020, and from Brazil in 2020 revealed that the MLL-F super-clone persists in the U.S. and is present throughout Brazil. The utilization of multiple sources of resistant sorghum and use of insecticides with differing modes of action may be beneficial to prevent the development of new biotypes and insecticide resistant genotypes of *M. sorghi*. 

## Data Availability

Raw fragment data can be found in Supplemental Table S2 and analyzed data can be found in the figures in this paper.

## Supplementary Materials

The following supporting information can be downloaded at: https://www.mdpi.com/article/10.3390/insects13050416/s1. Table S1: *Melanaphis* spp. and *Sipha flava* collection information for samples collected in 2019 from the United States, 2020 from the United States and Brazil, and samples used as the controls. Table S2: Allele sizes of 2019 and 2020 *Melanaphis sorghi* samples collected in the United States or Brazil.
